# The Biomedical Applications of Biomolecule Integrated Biosensors for Cell Monitoring

**DOI:** 10.3390/ijms25126336

**Published:** 2024-06-07

**Authors:** Kyeongseok Song, Soon-Jin Hwang, Yangwon Jeon, Youngdae Yoon

**Affiliations:** Department of Environmental Health Science, Konkuk University, Seoul 05029, Republic of Korea; kssong1999@konkuk.ac.kr (K.S.); sjhwang@konkuk.ac.kr (S.-J.H.);

**Keywords:** antibody, aptamers, biosensors, cell monitoring, chemical sensors, enzymes

## Abstract

Cell monitoring is essential for understanding the physiological conditions and cell abnormalities induced by various stimuli, such as stress factors, microbial invasion, and diseases. Currently, various techniques for detecting cell abnormalities and metabolites originating from specific cells are employed to obtain information on cells in terms of human health. Although the states of cells have traditionally been accessed using instrument-based analysis, this has been replaced by various sensor systems equipped with new materials and technologies. Various sensor systems have been developed for monitoring cells by recognizing biological markers such as proteins on cell surfaces, components on plasma membranes, secreted metabolites, and DNA sequences. Sensor systems are classified into subclasses, such as chemical sensors and biosensors, based on the components used to recognize the targets. In this review, we aim to outline the fundamental principles of sensor systems used for monitoring cells, encompassing both biosensors and chemical sensors. Specifically, we focus on biosensing systems in terms of the types of sensing and signal-transducing elements and introduce recent advancements and applications of biosensors. Finally, we address the present challenges in biosensor systems and the prospects that should be considered to enhance biosensor performance. Although this review covers the application of biosensors for monitoring cells, we believe that it can provide valuable insights for researchers and general readers interested in the advancements of biosensing and its further applications in biomedical fields.

## 1. Introduction

A sensor is simply described as a device that converts various inputs into digitized output signals. Many different types of sensors have been developed and used to monitor and detect these inputs. A sensor was defined by Fraden et al. as “a transducer that receives an input signal or stimulus and responds with an electrical signal bearing a known relationship to the input” [1]. To function as a sensor, it is necessary to possess an element for detecting (or sensing) various inputs and an element to convert the input into electric signals. In fact, sensing and signal-transducing elements are the key components that make up sensors, including chemical sensors and biosensors.

With rapid advances in several scientific fields, sensing and signal-transducing elements have been diversified and upgraded. Sensors are categorized according to the types of sensing or signal-transducing elements [2,3]. Moreover, integrating interdisciplinary sciences has contributed to diversifying the types of sensing and signal-transducing elements, making it challenging to categorize sensors into certain groups. In this regard, there are numerous review papers on various sensor types, which are further subdivided into chemical sensors and biosensors [4,5,6,7,8,9]. These reviews have covered various sensors depending on the materials consisting of sensing elements, such as graphene, carbon nanotubes, and biomolecules; similarly, they have also covered sensors based on the types of signal-transducing elements such as fiber optic-based, electrochemical, microfluidic-based, and surface plasmon resonance-based sensors. Rapid advances in nanomaterial sciences, miniaturization, and fabrication technologies have substantially enhanced the capabilities of sensor systems.

Typically, sensors are designed to detect and monitor targets that adversely impact human health and environmental systems. Monitoring the levels of specific targets and assessing their associated risks are crucial processes for safeguarding human health. In this regard, a wide variety of chemical sensors and biosensors have been developed and applied for detecting harmful materials. In particular, sensors for environmental toxicants, such as heavy metals, toxins, chemicals, and toxic gases, have been actively investigated. For example, diverse sensors, including transcription factor (TF)-based biosensors, optical sensors, and nanostructured sensors, have been developed and applied to monitor heavy metals [10,11,12,13]. Similar to detecting toxic materials, it is important to monitor pathogenic microorganisms to secure human health; moreover, various chemical sensors and biosensor systems have been reported for this purpose [14,15].

To date, sensors, including chemical sensors and biosensors for detecting toxic materials, have been actively investigated to secure human health. Simultaneously, scientific advancements in diverse research fields have greatly enhanced the methods for sensing targets. The application fields were further broadened in these respects, and cells became targets for various sensors because cells could indicate or communicate a great deal of information. Furthermore, sensors targeting cells have been extensively investigated in recent times because cells can represent illnesses, abnormalities, functions, and infections. For this reason, the application fields of sensors expanded to the medical field for early diagnosis of human diseases. Since diseases such as cancers induce abnormalities in cells and produce unique biomarkers, sensor systems to detect those targets with high precision and sensitivity could be used as a tool for early diagnosis. In this regard, cell monitoring could play a crucial role in health monitoring and medical diagnostics [16,17].

As described above, sensor systems have been actively developed and applied to monitor and detect various targets. With the rapid advance in diverse research fields, a large number of target sensing elements and signal-transducing elements have been developed, thereby leading to the emergence of new types of sensors. In terms of this aspect, here, we discuss biosensing systems for monitoring cells to gather information and evaluate the conditions of cells. Among the different types of biosensor systems, we focus on biosensors based on chemical sensor systems integrating biomolecules as components and their applications related to securing human health. Finally, we address the present challenges in biosensor systems and the future prospects that should be considered to enhance biosensor performance. Although this review only covers a limited portion of biosensors used for gathering information about cell conditions, we believe that it should be valuable for researchers and general readers interested in the advancements of biosensing systems and their applications for the early detection of abnormalities in cells.

## 2. Cell Monitoring for Biomedical Applications

Prior to examining various sensor systems for cell monitoring, it is essential to address the necessity and importance of cell monitoring. Because the states (or conditions) of cells reveal a large amount of information, the importance of cell monitoring has been highlighted in various fields, including bioengineering, the food and environmental industries, and the medical sector, in particular. Living organisms experience various biological and physiological changes due to external stresses, pathogen invasions, and abnormalities caused by diseases. Thus, it is critical to detect those cellular changes for cell monitoring. Diagnostics based on antibody detection would be one example of cell monitoring. Cell abnormalities, such as cancers and pathogen invasion, induce the production of specific antibodies as responses, and the states of cells could be discovered by detecting antibodies. Conclusively, cell monitoring can be achieved by detecting the responses induced by external stimuli, thereby providing valuable information for making decisions on the condition of cells. Moreover, it should be noted that monitoring of cells is the detection of biological markers originating from cells as well as direct cell detection. However, the former is relevant for target detection by sensor systems.

To do this, consideration should be given to what could serve as an indicator of cellular states and how alterations at the cellular level might be detected, especially for the diagnosis of human disease. All living cells generate various types of signals, including electrochemical and optical changes on their surface, in response to environmental changes [18,19]. In addition, cells display different cellular membrane compositions and secrete proteins and metabolites derived from various cellular processes [20,21,22]. Therefore, it is inferred that the techniques for detecting cellular changes can be employed as sensing systems for biomedical applications.

## 3. Chemical Sensor Systems for Cell Monitoring

Before going over the biosensing systems, it is necessary to discuss the chemical sensor systems. Classically, a chemical sensor is defined as “a device that transforms chemical information into an analytically useful signal” [23]. This information may originate from a chemical reaction of the analyte or from the physical properties of the system being investigated [23]. Sensors, including chemical sensors, can be classified as optical, electrochemical, electrical, magnetic, or thermometric sensors based on their operating principles. Most of all, these chemical sensor systems have been used as platforms for the detection of various materials [24,25]. Integrating cell-recognizing elements into these platforms provides a chemical sensor capable of monitoring cells. Therefore, it is important to have specific target and sensing elements that can indicate the states of cells to generate sensors for cell monitoring.

Among the chemical sensors, we focused on electrochemical sensors, one of the most utilized chemical sensors for cell monitoring, because the signals from cells in response to their cellular conditions induce electrochemical changes such as potential or current on the surface of signal transducers. Deshpande et al. [26] reviewed the redox potentials of reactive oxygen and nitrogen species (ROS/RNS) in cells that were monitored using diverse electrodes and nanomaterial-conjugated electrodes. Electrochemical techniques were used to monitor ROS/RNS by employing electrodes fabricated with various materials, including metal nanoparticles (metal NPs), quantum dots (QDs), graphene oxide, and chemicals. Similarly, Yoshinobu et al. [27] reported the application of a light-addressable potentiometric sensor (LAPS) for cell monitoring. The LAPS was used to quantify the acidification rate for cell monitoring by measuring the changes in AC photocurrent, extracellular potential, and pH via appropriate probes functioning as signal transducers. Similar to the aforementioned electrochemical sensors, the application fields of LAPSs have expanded along with the advancement of sensing elements. The LAPS was used to detect *E. coli* in orange juice and acidic metabolites in breast cancer cells by integrating it with pH-sensitive hydrogen nanofiber and microfluidics, respectively [28,29]. In addition to these applications, the LAPS was used as a biosensing platform for cell monitoring by integrating it with enzymes, DNA, and aptamers [30,31]. Most of all, the integration of biomolecules into chemical sensor systems gives enhanced target selectivity by direct interaction between targets and biomolecules. For example, Li et al. reported that the aptamer-integrated LAPS detects alpha-fetoprotein (AFP) by the interaction between AFP and aptamer with 0.1–100 μg/mL of detection ranges [30]. Moreover, the LAPS employing a glucose oxidase/reduced graphene oxide–chitosan–ferrooxidase/gold nanoparticles (GOX/RGO-CS-Fc/AuNPs) fabricated electrode surface was capable of detecting glucose in human serum samples by the interaction between GOX and glucose with 0.01–4.00 mg/mL of detection ranges and 0.001 mg/mL of detection limit [32].

**Figure 1:**
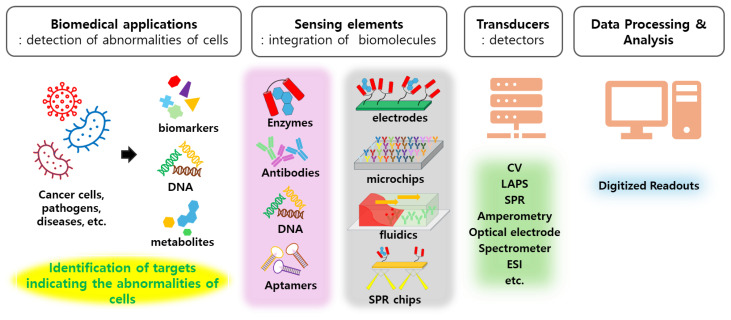
The illustration of common structures of biosensors employing chemical sensor systems as platforms. The targets originated from cells were interacted with appropriate biomolecules integrated into sensing elements, and the interactions were transmitted as signals to transducing elements used in chemical sensor systems.

To the same extent, biosensors could be obtained by integrating biomolecules as components of chemical sensor systems because they share the basic principles except for the inclusion of biomolecules (Figure 1).

Here, several chemical sensors, including electrochemical, optical, and fluorescent chemical sensors for monitoring cells and cellular changes, are listed in Table 1, along with the targets, sensing elements, signal-transducing methods, and detection ranges. In addition to the electrochemical sensors mentioned above, an optical sensor employing TiO_2_/Au hybrid film as a sensing element was reported to diagnose breast cancers by monitoring formaldehyde [33]. Moreover, fluorescent materials such as quantum dots (QDs) and carbon dots (CDs) were used to detect diseases by monitoring the biomarkers indicating specific diseases [34,35]. However, they might be considered fluorescent probes rather than sensors because of the lack of signal-transuding elements. Nonetheless, fluorescent probes such as QDs, CDs, chemical dyes, and metal NPs were applied to monitor cells or to diagnose diseases by employing fluorescence instruments as signal-transducing devices [36,37].

In addition to these chemical sensors, various types of chemical sensors employing combinations of different sensing elements such as metal–oxide–semiconductors (CMOSs), carbon nanotubes (CNTs), NPs and chemicals, and signal-transducing elements such as nanomaterial fabricated electrodes, LAPS, surface plasmon resonance (SPR), Raman spectroscopy, optic fiber, fluorescent spectrometry, and microfluidics to detect cells and diseases have been reported [38,39,40,41]. Moreover, new materials for target sensing are continuously being developed and applied to these sensor platforms due to the rapid advances in nanomaterials (NMs) and fabrication technologies. Currently, advances in chemical sensors are driving the development of biosensors by integrating biomolecules into these sensor systems. Therefore, advancements in chemical sensors are critical for the development of biosensors in terms of providing platforms for biosensors.

**Table 1:** Chemical sensors for cell monitoring.

Targets	Sensing Elements	Signal Transducers	LOD/ Linear Range	Targets/Application	Ref.
Acidification level	pH-sensitive hydrogel nanofiber	LAPS	10^2^ CFU/mL	*E. coli*/*S. typhi*	[28]
Acidification level	Silicon oxide/silicon nitride layer	LAPS- microphysiometer	-	Human breast cancer cells MCF-7	[29]
Formaldehyde	TiO_2_/Au hybrid film	SPR	0.2 ppm/ 0.2–1.8 ppm	Breast cancer	[33]
Alkaline phosphatase	ZnSe/ZnS QDs	Fluorescence spectrometry	0.57 U/L 4–96 U/L	Chronic kidney disease	[34]
β-glucuronidase	Fluorescent nitrogen-doped CDs	Fluorescence spectrometry	0.3 U/L 1 to 15 U/L	Early diagnosis of cancer	[35]
NAG β-galactosidase	Silicon nanoparticles (SiNPs)	fluorometric/colorimetric analysis	0.66 U/L 13.1 U/L	Kidney diseases diagnosis	[42]
Isoprene	Prism/Au/air cavity/(GaN/SiO_2_)^10^	Tamm plasmon resonance	80 ppb 0–600 ppb	Chronic liver fibrosis	[43]
Norepinephrine	Pt surface electrodes on CMOS microchip	trans-impedance amplifier	8–1024 µM	Electrochemical Imaging of live tissue	[44]
NO/nitric oxide	Au/RGO-TiO_2_-ITO electrode	CV	5 nM/ 20–500 nM	HUVECs	[45]
H_2_O_2_/oxidative stress	Au-Pd alloy NPs/graphene QDs	Amperometry	500 nM	Breast cancer cells	[46]
Glucose	Ni_3_C/Ni nanochain modified electrode	CV	0.28 μM/ 1.0–6.5 μM	Biological fluids/clinical application	[47]
Cells	Au/Cr coated glass	EC-SPR	-	Monitoring liver cancer cell viability	[48]

QDs: quantum dots; RGO: reduced graphene oxide; ITO: indium–tin oxide; CV: cyclic voltammetry; NAG: N-acetyl-β-D-glucosaminidase; HUVEC: primary human umbilical vein endothelial cell; LAPS: Light-addressable potentiometric sensor; EC-SPR: electro-chemical-surface plasmon resonance.

## 4. Biosensor Systems for Cell Monitoring

### 4.1. Chemical Sensor Systems as Platforms for Biosensors

Biosensors are generally defined as “chemical sensors in which the recognition system utilizes a biochemical mechanism” or “devices that transform the interactions between bioreceptors and analytes into a logical signal proportional to the concentration of reactants” [49,50]. From these two definitions, it is evident that a biosensor is a device that detects signals by using biomolecules as sensing elements for targets. In conclusion, biosensors must have biological molecules as sensor components and transduce signals originating from biological mechanisms. Signals originating from biological mechanisms, such as cellular metabolites, proteins, antigens, and ions, are diverse. Biomolecules, such as DNA, enzymes, and antibodies, can function as sensing elements in sensor systems. As shown in Figure 1, diverse biosensors could be generated by integrating biomolecules with a combination of chemical sensor systems and applied to detect and monitor targets for their own purposes. Biomolecules such as enzymes, antibodies, DNA, and aptamers were integrated as components of sensing elements to detect targets such as metabolites and biomarkers indicating the states of cells. Then, the signals induced by the interactions between biomolecules and targets were transmitted to outputs by diverse transducers, including electrodes, LAPS, CV, amperometry, and SPR. Here, we would like to emphasize that the basic principle of biosensors is the same as that of chemical sensors and chemical sensor systems could be platforms for biosensors.

### 4.2. Biomolecule Integrated Biosensor Systems

#### 4.2.1. Enzyme-Based Biosensors

The concept of an enzyme-based biosensor was presented in the 1960s, marking the development of the earliest biosensor in the modern era [51]. Enzymes are often used as components of sensors because of their capacity to recognize specific targets and their capability to generate signals. Typically, enzymes, such as oxidases, reductases, and cholinesterases, are employed as sensing elements in sensors because their activities indicate the concentration of targets [52,53,54]. One of the challenges in the construction of enzyme-based biosensors is to ensure that the integrated enzymes in the sensor systems remain stable and functional. However, this challenge can be overcome by incorporating enzymes into NMs and hydrogels. Subsequently, enzymes can be incorporated into biosensors by integrating sensing and signal-transducing elements, including carbon nanotubes (CNTs), metal NPs, microfluidic chips, optic fiber, and electrodes [55,56,57]. Generally, enzyme-based biosensors were developed to target inhibitors that repress enzyme activity because of the inherent nature of enzymes. Because there are numerous reviews on enzyme-based biosensors that target toxic materials [56,58,59], we focused on recent studies on enzyme-based biosensors and their potential for biomedical applications.

**Figure 2:**
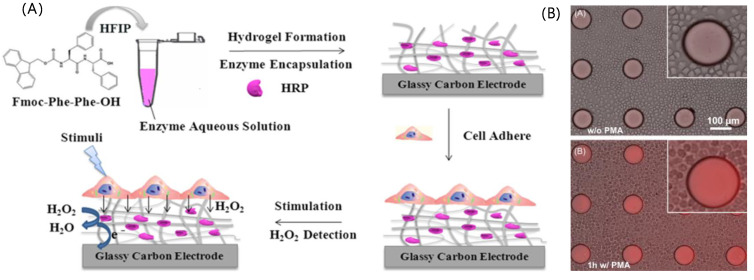
Enzyme-based biosensors for monitoring cells. (**A**) Schematic illustration for the construction of biointerface toward enzyme-based electrochemical biosensors and cell monitoring, (**B**) fluorescence images of macrophages adherent around HRP-containing PEG microstructure with PMA (up) and without PMA (down) treatments. The images (**A**,**B**) were reprinted from [60] and [61], respectively.

Enzymes serve as sensing elements that detect signals from cells or reporters while simultaneously converting signals. Measuring signals such as H_2_O_2_ and glucose can provide insights into cellular states, such as the presence of oxidative stress and diabetes. Lian et al. reported horseradish peroxidase (HRP)-based electrochemical sensors for monitoring H_2_O_2_ in HeLa cells [60]. In the case of enzyme-based biosensors, the stability of the enzyme is critical for the performance of biosensors and an obstacle to solve. In this regard, they constructed a bio-interface on glassy carbon electrodes (GCEs) by combining HRP with a self-assembled peptide nanofibrous hydrogel composed of *N*-fluorenyl methoxycarbonyl-diphenylalanine (Fmoc-FF) (Figure 2A). They demonstrated the performance of the biosensors by measuring the H_2_O_2_ released from HeLa cells on an HRP/Fmoc-FF hydrogel-modified electrode at a low detection limit of 18 nM. Similarly, another research group reported enzyme-based biosensors to monitor H_2_O_2_ in cells by incorporating HRP into polyethylene glycol (PEG) hydrogels [61]. They fabricated hydrogel micropatterns on glass plates and detected H_2_O_2_ released from cells by measuring fluorescence signals (Figure 2B). Matharu et al. developed a biosensor to monitor oxidative stress in primary hepatocytes using HRP/PEG hydrogels/AuNP electrode, which was monitored by CV with detection ranges of 0.29–1.16 μM [62]. Although they did not demonstrate the application of these biosensors in practical fields, the biosensors would be applied to medical fields for early diagnosis and monitoring of diseases related to oxidative stresses and glucose levels [63,64].

In addition to hydrogels, diverse nanomaterials, including CNTs, graphene, QDs, and metal NPs, have been widely used to stabilize and incorporate enzymes as biosensor components. Enzymes, such as acetylcholinesterases, glucose oxidases (GOx), and HRPs, are integrated (or embedded/encapsulated) into NMs to serve as biosensor components. Based on the characteristics of NMs, the signals generated by enzymes are monitored using various signal-transducing elements, such as CV, chronoamperometry, and potentiometry. Rassas et al. [65] reported glucose biosensors that utilized glucose oxidase encapsulated in a chitosan–*k*–carrageenan polyelectrolyte complex. The chitosan/Gox complex biosensor, which was fabricated using gold electrodes, exhibited a wide linearity range of 5–7 mM glucose. Moreover, many research groups have developed GOx-based glucose sensors by integrating NMs, such as CNTs and AuNP-decorated graphene–CNTs [66,67]. Moreover, glutathione peroxidase (GSH-Px) has been used as a sensing element to monitor glutathione levels in biological body fluids. An enzyme/graphene oxide (GO)/nafion complex integrated on GCE was used for monitoring GSH over a range of 0.003–370.0 μM with a detection limit of 1.5 nM using differential pulse voltammetry (DPV) [68]. Similarly, Khan et al. have reported oxidases-based electrochemical sensors employing glucose oxidase, lactate oxidase, and xanthine oxidase linked to MXCeO_2_ (MXene-based 2D nanostructure decorated with enzyme mimetic cerium oxide nanoparticle) [69]. In this biosensor system, MXCeO_2_ acts as a novel platform for enzymes and as a catalytic amplifier for electrodes to enhance the sensitivity to glucose, lactate, and hypoxanthine. Conclusively, it was inferred that the new enzyme-based biosensors could be generated if appropriate techniques were used to stabilize enzymes and to integrate enzymes to the components of sensor systems.

Although the targets of enzyme-based biosensors are limited compared to those of other biosensors, their medical applications have continued to increase because of their straightforward and rapid detection of biomarkers, such as glucose, glutathione, and H_2_O_2_, indicating abnormalities of cells. Since the shortcomings of enzyme-based biosensors are compensated by the rapid advances in technologies, it would be promising to enlarge the applications of enzyme-based biosensors. The enzyme-based biosensors mentioned in this section are listed in Table 2, along with sensing elements, signal transducers, detection ranges, and applications.

#### 4.2.2. Antibody-Based Biosensors

Antibodies are powerful tools for diagnostic applications owing to their target-sensing capabilities. Similar to enzyme-based biosensors, antibody-based biosensors employ antibodies for the biorecognition of targets. The process involves transferring the interaction between antibodies and targets into output signals using signal transducers such as electrodes. Nevertheless, antibody-based biosensors are superior to enzyme-based biosensors in terms of target diversity. In contrast to enzymes, the target specificity of antibodies can be manipulated to generate various biomolecule-targeting antibodies. Therefore, antibody-based biosensors can be used in diagnosis to identify biomarkers that indicate specific diseases and pathogens. Owing to the nature of antibodies, they are ideal materials for sensing elements in biosensor systems. Generally, antibody-based biosensors (i.e., immunosensors) are used to monitor specific antigens [70]. Various detection methods, including reflectometric interference spectroscopy, SPR, enzyme-linked immunosorbent assay (ELISA), amperometry, voltammetry, potentiometry, and conductometry, have been employed as signal-transducing elements for antibody-based biosensors [71,72].

**Figure 3:**
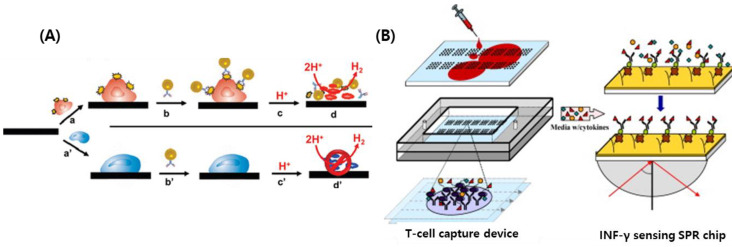
Antibody-based biosensors for monitoring cells. (**A**) Antibody-modified AuNPs catalyze the hydrogen ion reduction selectively on HMy2 cells, (**B**) schematic illustration of biosensors employing anti-CD4 antibody as a sensing element and SPR as a transducing element to detect IFN-γ for diagnosis. The images (**A**,**B**) were reprinted from [74] and [75], respectively.

Although the basic principles of antibody-based biosensors are the same, new types of biosensors have been intensively studied. Similar to enzyme-based biosensors, the development of antibody-based biosensors has been accelerated by rapid advances in material sciences, miniaturization, and fabrication techniques. In this study, we reviewed recent studies employing new and interesting technologies for antibody-based biosensors and their applications. Sekhar et al. reported an antibody-based biosensor that employed silica nanowires (SNWs) as templates for linking antibodies. This biosensor was used to detect interleukin-10, a biomarker for lung cancer, by measuring the optical response of the immunoassay [73]. The monitoring and quantification of tumor cells, HMy2 lines, have also been conducted using an AuNP-αDR antibody coupled with electrochemical sensors [74]. The AuNP/antibody specifically interacted with tumor cells that express LA-DR on their surface, generating signals with a detection limit of 4000 cells/700 μL (Figure 3A). Similar to this study, human CD4 T cells were monitored by detecting interferon-gamma (IFN-gamma) using SPR as a signal transducer [75]. They employed monoclonal IFN-gamma antibody-integrated SPR chips as sensing elements to determine changes in the reflective index induced by INF-gamma binding (Figure 3B). Interestingly, compared to other biomolecules, antibodies have been employed in micro- and nano-technology-based assays. Recent progress in microfabrication technology and the construction of microstructure-, chip-, and microfluidic-based assays have emerged with the integration of antibodies as sensing elements [76]. Washburn et al. developed microchip-based sensors for the detection of cancer biomarkers [77]. Since these chips were functionalized with DNA and conjugated to specific antibodies for cancer biomarkers, diverse cancers could be diagnosed by measuring optical changes in a microfluidic device. Overall, the microfluidic-based assay is considered a powerful tool for “point-of-care” (POC), and has been employed not only for antibody-based biosensor systems but also for diverse types of biosensors as signal transducers [78,79].

Like enzyme-based biosensors, it is critical to make antibodies stable and to integrate antibodies into sensor systems to obtain antibody-based biosensors. However, antibodies are more versatile than enzymes by means of stability and the ranges of targets. When the targets, including metabolites and biomarkers indicating abnormalities of cells and corresponding antibodies, are available, new biosensors could be constructed by integrating them into different types of chemical sensor systems. In this regard, antibody-integrated NPs, NMs, graphene, and microfluidic chips were employed as sensing elements of diverse sensor platforms to be antibody-based biosensors [76,80,81,82]. As an example, Wallace et al. reported an antibody-based electrochemical sensor to detect the biomarkers, transactive response DNA binding protein (TDP-43), for amyotrophic lateral sclerosis [83]. They put antibodies on the Au surfaces of electrodes and detected TDP-43 by EIS with an 11 ± 6 nM detection limit. In addition to these studies, antibody-based biosensors have been actively applied as diagnostic tools in biomedical fields.

As listed in Table 2, antibody-based biosensors were applied to detect biomarkers and metabolites related to diseases such as diverse cancers, sickle cell disease, and COVID, more than other types of biosensors. Therefore, the potential of antibody-based biosensors for diagnosing diseases would be increased along with finding novel biomarkers indicating specific diseases.

#### 4.2.3. DNA Hybridization-Based Biosensors

DNA-based biosensors have been extensively researched in the environmental, medical, and food industries owing to their simplicity, stability, biocompatibility, and cost-effectiveness, which originate from their well-understood and straightforward hybridization mechanism. However, DNA-based biosensors are not actively applied to monitor diseases because of the nature of sensing mechanisms. Similar to the biosensors mentioned above, DNA is used as a sensing element in various types of existing sensor systems, and the target interaction with DNA is transduced as output signals. Watts et al. developed an optical biosensor to detect *Legionella pneumophila* using a specific oligomer-coated SPR system with a 9.2 nM detection limit toward 40-mer of target DNA [84]. Although DNA hybridization is a powerful tool for detecting target DNA, it may be the weakest aspect of DNA hybridization-based biosensors when it comes to preparing DNA from the targets of interest. However, DNA hybridization-based biosensors have been developed in conjunction with new findings in materials, fabrication technologies, and signal transducers and applied for the diagnosis of various diseases [85,86].

Similar to enzyme- and antibody-based biosensors, target-specific DNA (probes) was implanted into sensing elements in various chemical sensor systems; the hybridization-induced signals, such as the changes in electrochemical properties, were detected by transducers in sensor systems [87]. The COVID-19 diagnosis was a good example of the application of DNA-based biosensors. Tripathy et al. reported the development of a miniaturized device equipped with DNA probe-conjugated AuNP/Ti electrodes for COVID-19 diagnosis [88]. Hwang et al. also reported DNA-based biosensors for COVID-19 employing the immobilizing DNA on a glass wafer on an interdigitated electrode (IDE) and an impedance analyzer as sensing and signal transducing elements [89].

Basically, DNA hybridization-based biosensors detect target DNA through complementary hybridization, making them highly specific to targets when employing DNA probes. Although it is widely accepted as a powerful tool for detecting microorganisms in environmental systems, it is less practical for cell monitoring in biomedical fields because of the limited amount of target DNA in human samples. However, the disadvantages associated with DNA-based biosensors have been overcome by employing other types of DNA molecules as sensing elements for sensor systems, such as DNAzymes and aptamers, which possess target selectivity [90,91]. As mentioned above, the application field of biosensors based on DNA hybridization is limited to detecting microorganisms rather than human diseases, but the versatility of DNA is adapted as sensing elements of aptamer-based biosensors, thereby enhancing the application fields. Additionally, advances in signal amplification techniques such as hybridization chain reaction (HCR), enzyme-assisted target recycling (EATR), and rolling circle amplification (RCA) also contribute to improving the performance and enlarging the application area of DNA-based biosensors [92,93,94].

#### 4.2.4. Aptamer-Based Biosensors

Aptamers are single-stranded oligonucleotides (DNA, RNA, or modified nucleotides) that interact with target elements with high affinity. In contrast to conventional DNA-based biosensors, aptamers exhibit a distinct 3D structure and tend to interact with target biomolecules as a “lock-and-key” model [95]. Aptamer-based biosensors, known as aptasensors, have garnered interest because of their high specificity, sensitivity, real-time monitoring ability, and versatility achieved through the systematic evolution of ligands by exponential enrichment (SELEX) [96]. The conventional development of the SELEX protocol includes multiple cycles of (a) generation of a large random RNA library; (b) immobilization onto a carrier; (c) large-scale target interaction assay, separation, and washing; (d) amplification; and (e) pool conditioning [97]. Although the SELEX protocol is considered costly, time-consuming, and labor-intensive owing to its complexity, repeatability, and uncertain selection process, aptasensors are superior to other bioreceptor-based sensors in terms of cost because they are composed of oligonucleotides, which possess a lower weight and reduced cost and can be synthesized in vitro [98,99,100]. Most importantly, the target selectivity of aptamers can be designed and modulated according to their targets, including proteins and small molecules.

Like other biosensing molecules, interaction signals between aptamer and target molecules can be transduced into acoustic, optical, colorimetric, and electrochemical signals. In this regard, there have been several review articles on aptamer-based biosensors incorporating NPs in electrochemical sensors, nanofabricated chips in microfluidic-based sensors, and NMs in optical sensors [101,102,103]. Moreover, Han et al. [104] summarized the recent findings on aptamer-based biosensors, along with the strategies to design biosensors and the working modes of aptamers in biosensors. The roles of aptamers in biosensors are similar to those of antibodies. However, the advantages of aptamers over antibodies, such as chemical stability, ease of chemical modification, relative ease of synthesis, biocompatibility, cost-effectiveness, and ease of integration with other materials, have accelerated the application of aptamer-based biosensors.

Over the past few decades, numerous studies have reported the detection of proteins, small molecules, and environmental toxins using aptamer-based biosensors [105,106]. Among the diverse applications of aptamer-based biosensors, this review focused on medical and therapeutic applications based on the monitoring of pathogens. Recently, several research groups reported the application of aptamer-based biosensors for the detection of pathogenic bacteria, such as *Salmonella*, *Escherichia coli*, *Pseudomonas aeruginosa*, and *Vibrio*, in food products. *Salmonella* was detected using AuNP-conjugated aptasensors with a cell count in the range of 10^4^–10^5^ copies [107]. However, because of the absence of signal-transducing elements, they might not qualify as biosensors; therefore, they are referred to as AuNP-based aptasensors. In contrast, Shahrokhian et al. developed an electrochemical aptamer-based biosensor for detecting *E. coli* [108]. In this study, aptamers were fabricated on a surface-modified glassy carbon electrode, and the interactions between *E. coli* O157:H7 and the aptamers were detected by DPV. Similarly, Shin et al. developed an aptamer-based biosensor to detect *Vibrio* using SPR [109]. They verified the effectiveness of the aptamer for *Vibrio* detection and then applied it to paper strip chips with a detection limit of 10^3^–10^4^ CFU/mL.

**Figure 4:**
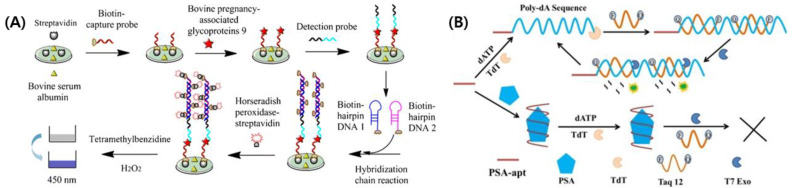
Diagrams for the working mechanisms of aptamer-based biosensors with HCR and EATR techniques. (**A**) The flowchart to detect bPAG9 using aptamer-based biosensors using HCR, (**B**) the principle of EATR-assisted detection using aptamer-based biosensors. The figures were reprinted from [116,117].

These days, the applications of aptamer-based biosensors have been moved to biomedical fields such as diagnostics and POCs [98,110,111]. As mentioned for antibody-based biosensors, different metabolites and biomarkers indicating the state of cells are targeted for aptamer-based biosensors because aptamers play a role of sensing elements similar to antibodies. Recent reports indicate that a microfluidic aptamer-based electrochemical biosensing platform was developed for monitoring cardiac organoid damage [112]. Because damaged cardiac tissue released creatine kinase MB (CK-MB), they used microfluidic chips incorporating a CK-MB sensing aptamer as a sensing element in conjunction with an Au microelectrode at a 2.4 pg/mL detection limit. By employing aptamers as antigen receptors, it was possible to monitor the cellular damage caused by drug treatments. Similarly, Karpik et al. investigated an aptamer-based biosensor for detecting mucin1 (MUC1), a biomarker, in order to monitor prostate cancer cells [113]. The interaction between MUC1 and aptamer-coated Au electrodes was measured via square wave voltammetry (SWV) with a linear detection range of 0.65–100 ng/mL. Similar to these studies, there have been many excellent studies focusing on aptamer-based biosensors for monitoring cells and biomarkers [114,115]. In addition, target amplification techniques such as HCR and EATR were applied to enhance the performance of aptamer-based biosensors. Lu et al. developed an aptamer-based biosensor specific for bovine pregnancy-associated glycoprotein 9 (bPAG) and applied an HCR strategy [116]. Notably, HCR was directly applied to the developed aptamer-based biosensors and contributed to signal amplification (Figure 4A). Moreover, aptamer-based biosensors can facilitate EATR. Chen et al. developed an aptamer-based biosensor capable of sensing prostate-specific antigens (PSA), which are considered biomarkers for prostate cancer [117]. They improved the detection range of biosensors by combining terminal deoxynucleotidyl transferase (TdT) and T7 exonuclease-based EATR-assisted and aptamer-based biosensors (Figure 4B).

In summary, aptamers are fascinating molecules for sensing elements in sensor systems owing to their physical and biological nature. Aptamers play roles as sensing elements like antibodies and as both sensing elements and linkers to the components in signal transducing elements. Thus, the findings of appropriate aptamers targeting biomarkers indicating the states of cells would lead to the development of new biosensors by integrating the sensor systems as components. Because of the versatility of aptamers, aptamer-based biosensors have been continuously advancing by incorporating new materials and technologies while being applied in various fields.

**Table 2:** Biomolecule-based biosensors for cell monitoring.

	Target	Sensing Elements	Signal Transducers	LOD/ Linear Range	Applications	Ref.
Enzyme	H_2_O_2_	HRP/Fmoc-FF modified electrode	CV	18 nM	HeLa cells	[60]
	H_2_O_2_	HRP/PEG-hydrogels AuNP electrode	CV	0.29–1.16 μM	Hepatocytes	[62]
	glucose	chitosan/Gox complex-Au electrode	SWV	5 μM/ 5 μM–7 mM	Saliva samples	[65]
	glutathione	GSH-Px-GO/nafion/GCE	DPV	1.5 nM/ 0.003–370.0 μM	Body fluids	[68]
	Glucose Lactate hypoxanthine	Oxidase/MXCeO_2_	Electrodes	0.49 μM 3.6 μM 1.7 μM	Artificial sweat	[69]
	Acetylcholine	ACHE-conjugated Au electrode	EIS	5.5–550 μM	Rat brain slurry Rat whole blood	[118]
	L-MC-LR	MlrB-MWCNT/GCE	CV/EIS	0.127 pg/mL 0.001–100 ng/mL	Water samples	[119]
	*E. coli* *S. aureus*	β-galactosidase-AuNPs	CV	100 CFU/mL	Water samples	[120]
antibody	Interleukin-10	Antibody-SNW	Spectrometer	100 μg/mL	Monitoring lung cancer	[73]
	TDP-43	Antibody-Au electrode	EIS	11 ± 6 nM	Amyotrophic lateral sclerosis	[83]
	Hemoglobin	Antibody-microfluidics	Plate reader	4.0 g/L for Hb A 5.0 g/L for Hb S	Monitoring sickle cell disease	[121]
	Biomarkers for cancers	Antibody-conjugated microchips	SPR	-	Diagnosis for diverse cancers	[77]
	COVID-19 Spike S1	S1 antigen-RGO nanoflakes	CV	2.8–16.9 fM	COVID diagnosis	[81]
	Cytokines	Antibody-ssDNA on chips	Plate reader	1 fg/mL 1 fg/mL–1 ng/mL	Health monitoring	[122]
	Cells	Anti-EpCAM-GO-COOH	LAPS	10 cells/mL	Circulating tumor cells	[123]
	*E. coli* O157 *Salmonella*	Antibody-coated graphite felt electrode	OSWV	400 cells/mL	Detecting pathogens	[124]
DNA	HER2, EpCAM, CD63	Au@Ag Nanocubes on AuFON	SERS	50 exosomes/mL	Human/bovine serum	[125]
	COVID-19 cDNA	DNA-IDE	Impedance analyzer	10 nM	COVID diagnosis	[89]
	Alpha-fetoprotein	Aptamer-AuNP	LAPS	92.0 ng/mL 0.1–100 μg/mL	Diagnosis of liver cancer	[30]
	*Salmonella*	AuNP-aptamer	Spectrometer	10^4^ to 10^5^ copies	Monitoring pathogens	[107]
	PSA	Aptamer-HCR-AuNP	Colorimetric	30 pg/mL	Human serum	[126]
	PSA	PSA-Aptamer/TdT/T7 Exo/Taq12	Fluorescence	0.043 pg/mL	Human serum	[117]

HRP: horseradish peroxidase; Fmoc-FF: N-fluorenyl methoxycarbonyl-diphenylalanine; SWV: square wave voltammetry; DPV: differential pulse voltammetry; Gox: glucose oxidases; GCE: glassy carbon electrode; GSH-Px: glutathione peroxidase; GO: graphene oxide; MXCeO_2_: MXene-based 2D nanostructure decorated with enzyme mimetic cerium oxide nanoparticle; ACHE: acetylcholinesterase; L-MC-LR: linear microcystin-LR; MWCNT: multi-walled carbon nanotubes; anti-EpCAM: anti-human epithelial cell adhesion molecule; OSWV: oster young square wave voltammetry; AuFON: hexagonal-packed gold film over nanosphere; SERS: surface-enhanced Raman scattering; PSA: prostate-specific antigen.

### 4.3. Recent Progresses in Biosensor Systems for Cell Monitoring

Diverse sensor systems, including chemical sensors and biosensors discussed above, share fundamental principles. Consequently, new types of sensors can be developed by integrating novel elements or technologies into existing systems. In addition to the previously mentioned biomolecules, advanced biological systems such as CRISPR (Clustered Regularly Interspaced Short Palindromic Repeats)/Cas (CRISPR-associated) systems and engineered cells (or tissues) have been employed (utilized) as target sensing and signal-producing elements in sensor systems [127,128]. With the emergence of new materials and nanofabrication technologies, biosensors based on these systems have rapidly expanded for biomedical applications.

CRISPR/Cas-based biosensors have been actively investigated and applied for diagnostic purposes [129,130,131]. Given that CRISPR/Cas is used for gene-editing, it can interact with target DNA/RNA sequences complementary to spacer sequences in CRISPR guide RNAs (gRNAs) [132]. By designing specific RNAs, the CRISPR/Cas/gRNA complex detects target sequences, resulting in the activation of the CRISPER/Cas system. Although the operational mechanisms of CRISPR/Cas biosensors vary depending on the types of Cas effectors and CRISPR/Cas systems, activated CRISPR/Cas can induce signals through cleavage-based and binding-based modes [128,133,134]. Thus, various types of CRISPR/Cas-based biosensors can be obtained by integrating targets in existing sensor systems [135,136,137]. Depending on the signals from targets, CRISPR/Cas-based sensors employ appropriate signal transducers to detect fluorescent, colorimetric, and electrochemical changes [138]. Since CRISPR/Cas biosensors target specific DNA sequences, identifying appropriate sequences for diagnosis is critical. Nonetheless, CRISPR/Cas biosensors are regarded as promising candidates for future diagnostic biosensing systems due to their superior sensitivity and selectivity.

Engineered cells and tissues can serve as sensing elements for monitoring signaling molecules in cellular environments. Advances in fabrication and nanotechnology have opened the door to engineering cell surfaces with aptamers, lipid-conjugated DNA, and nanomaterials, allowing these engineered cells to act as sensing elements to target detection [139,140,141]. Zhao et al. reported real-time monitoring of cellular environments using surface-engineered cells with aptamers that bind to platelet-derived growth factor (PDGF) and contain a pair of fluorescent dyes [142]. Although this approach necessitates an additional fluorescence microscope for monitoring cellular environments, efforts to integrate these systems into sensor platforms have resulted in the development of various types of biosensors based on engineered cells and tissues [143,144,145].

Here, we mention two systems as examples of recent progress. However, the evolution of biosensing systems has continued with developments in diverse research fields. The findings of new materials and technologies linked to advances in sensing and signal transducing elements, respectively, lead to the current progress in biosensing systems and enlarge their application fields.

## 5. Current Challenges of Biosensing Systems for Biomedical Applications

In terms of the biomedical application of biosensors, minimizing biofouling is critical for ensuring biosensor sensitivity and selectivity. Because biological media are rich in nonspecific proteins and biomolecules, electrochemical biosensor interfaces often suffer from biofouling caused by the nonspecific adsorption of biomolecules. Moreover, because clinically valuable biomolecules on the target primarily exist in trace amounts, biofouling can result in false positives and negatives. The importance of preventing biofouling is highlighted in subjects with clinically vulnerable patients, such as immunocompromised patients or those who have had prolonged hospital stays, because of the close association between biofilm formation and infections [146]. To minimize and delay biofouling in biosensor systems, hydrophilic, biomimetic, drug-eluting, and other materials have been investigated, and several anti-biofouling approaches have been suggested [147]. For example, the electrodes employed in electrochemical biosensors must be hydrophilic, electrostatically repulsive, and have smooth surfaces to prevent biofouling [148]. Because it is favorable for biosensor interfaces to be fabricated with highly hydrated materials to prevent biofouling, researchers conventionally use polymers, such as PEG, as hydration layers owing to their high hydrophilicity, nontoxicity, and biocompatibility [149,150]. In addition to these strategies, antifouling peptide hydrogel serves as a valuable tool for fabricating novel interfaces because of its biocompatibility and functionality to decrease biofouling [151].

The stability of biomolecules, such as proteins and antibodies, is another challenge associated with biosensors. As mentioned above, biomolecules, especially enzymes, employed as sensing elements need to be stable to ensure the sustainability of the biosensors. Enzyme stability is affected by environmental factors such as temperature, ionic atmosphere, pH, and humidity. Researchers have suggested the incorporation of various materials, such as hydrogels, nanoparticles, and electrolytes, to achieve enzyme stability in biosensors for storage and operation [152,153]. Nuclease-mediated degradation is an issue that must be overcome for DNA-based biosensor systems. Despite the cost-effectiveness and specificity of aptamers as sensing elements, they are susceptible to nuclease-mediated degradation in vivo; therefore, chemical modification of aptamers is inevitable [154]. One approach for preventing nuclease-mediated degradation is to utilize DNA enantiomers instead of d-DNA aptamers [155]. Moreover, aptamers can be modified during SELEX (in-SELEX) or after SELEX (post-SELEX) to protect the 2’ position and 3’ end, which are vulnerable to nuclease degradation [156]. To address the problem of aptamer stability, several research groups have suggested various modification strategies [157,158].

Aside from biofouling, stability, and nuclease-mediated degradation, there are numerous other challenges that need to be addressed to improve biosensors. Nonetheless, biosensor systems have many advantages over chemical sensors for monitoring cells and other targets. With the emergence of new technologies in different scientific fields, biosensors will be investigated more intensively and applied in diverse industries, especially in biomedical fields, in the near future.

## 6. Conclusions

Sensors, encompassing both chemical sensors and biosensors, are considered powerful tools for monitoring various targets, such as toxic materials, biomolecules, and cells. Sensors prioritize human health by monitoring harmful materials in environmental systems. Sensors initially focused on detecting harmful materials that posed a threat to environmental systems and human health, but their applications have expanded to include various purposes, such as health care, diagnosis, bioengineering, and synthetic biology. Over the past few decades, there has been an increasing demand for more rapid and straightforward procedures than conventional instrument-based methods for monitoring various targets. In this regard, various sensor systems have been used as alternative methods, and their performance has been improved to be similar to that of conventional methods. As outlined in this review, diverse sensor systems, including electrochemical and optical sensors utilizing different types of sensing and signal-transducing elements, have rapidly advanced with the emergence of new technologies.

Most of all, chemical sensor systems show superior sensitivity, short response time, and long-term stability depending on the types of sensing materials and signal transducers. However, they exhibit limited selectivity and specificity toward targets for cell monitoring. To address this issue, biomolecules such as enzymes, antibodies, DNA, and aptamers have been integrated into chemical sensor systems as sensing elements for biosensors. By integrating the biomolecules, the biosensors obtain the advantageous aspects of chemical sensors such as shorter response time and high sensitivity along with target selectivity. Of course, biomolecule-based biosensors possess several disadvantageous aspects, including poor stability of the sensing elements and biofouling, which must be addressed. Nonetheless, biosensor systems have been evolving with advances and convergences in diverse scientific areas. Like CRISPR/Cas systems and engineered cells and tissues, the targets and application fields of biosensor systems could be enlarged with the emergence of novel findings able to be integrated as components of sensor systems.

Although the purpose of cell monitoring differs from that of its application fields, it is most important to detect pathogens and abnormalities in cells to safeguard human health. As discussed above, various biosensors have been utilized in medical applications to detect biomarkers for human diseases, including prostate cancer, breast cancer, lung cancer, and diabetes, as well as pathogens, such as *E. coli*, *Vibrio*, and *Salmonella*, in human samples. Given the considerable potential of biosensor systems for disease diagnosis, efforts have been made to transition them to POC devices. Moreover, advances in nanotechnology have made it possible to integrate biosensor systems onto small devices, such as microchips and strips, and there have been several studies on portable and wearable POC devices. Although not yet operational in real-world settings, new POC devices that can diagnose multiple diseases will be developed along with the emergence of new materials, technology, and biosensor systems in the near future.

## Data Availability

Not applicable.
